# Silver Nanoflakes-Enhanced Anisotropic Hybrid Composites for Integratable Pressure Sensors

**DOI:** 10.3390/nano12224018

**Published:** 2022-11-16

**Authors:** Qingtian Zhang, Guolin Yun, Shida Jin, Zexin Chen, Shi-Yang Tang, Hongda Lu, Haiping Du, Weihua Li

**Affiliations:** 1 School of Mechanical, Materials, Mechatronic and Biomedical Engineering, University of Wollongong, Wollongong 2522, Australia; 2 Cambridge Graphene Centre, University of Cambridge, Cambridge CB3 0FA, UK; 3 Department of Electronic, Electrical and Systems Engineering, University of Birmingham, Edgbaston, Birmingham B15 2TT, UK; 4 School of Electronic, Computer and Telecommunications Engineering, University of Wollongong, Wollongong 2522, Australia

**Keywords:** conductive elastomer, anisotropic properties, highly sensitive pressure sensor, electronic skins

## Abstract

Flexible pressure sensors based on polymer elastomers filled with conductive fillers show great advantages in their applications in flexible electronic devices. However, integratable high-sensitivity pressure sensors remain understudied. This work improves the conductivity and sensitivity of PDMS-Fe/Ni piezoresistive composites by introducing silver flakes and magnetic-assisted alignment techniques. As secondary fillers, silver flakes with high aspect ratios enhance the conductive percolation network in composites. Meanwhile, a magnetic field aligns ferromagnetic particles to further improve the conductivity and sensitivity of composites. The resistivity of the composite decreases sharply by 1000 times within a tiny compression strain of 1%, indicating excellent sensing performance. On the basis of this, we demonstrate an integratable miniature pressure sensor with a small size (2 × 2 × 1 mm), high sensitivity (0.966 kPa^−1^), and wide sensing range (200 kPa). Finally, we develop a flexible E-skin system with 5 × 5 integratable sensor units to detect pressure distribution, which shows rapid real-time response, high resolution, and high sensitivity.

## 1. Introduction

Flexible pressure sensors have attracted extensive attention owing to their advantages in applications in soft robots [1,2], electronic skin [3,4,5], and wearable electronics [6,7,8]. As the vital components of most soft pressure sensors, flexible conductive composites have been vigorously developed in recent years. Generally, a flexible conductive composite is an elastomer matrix filled with conductive fillers, which endows the electrical conductivity from conductive fillers and the good mechanical properties from the polymer elastomer.

Most flexible conductive composites can be classified into capacitive [9,10,11], piezoelectric [12,13,14], and piezoresistive [8,10,15,16,17,18] types. Among them, piezoresistive composites tend to have a simple fabrication procedure, low cost, and comparatively low energy consumption when used in electronic devices. In the piezoresistive composite, the conductive fillers inside the matrix move under mechanical deformation, resulting in the creation or destruction of conductive paths, thus changing the resistance of the composite. Most conventional conductive elastomers are filled with only one type of conductive filler, such as carbon materials [19,20], metal materials [21,22], and conductive polymers [23,24], which are dispersed into elastic polymer matrices to form diverse conductive networks. However, single-filler composites often suffer from limitations in mechanical or electrical properties. The composite requires high filler contents to achieve electrical percolation [25], which not only impairs the flexibility and stretchability of the composite but also seriously hinders the cross-linking of the polymer matrix.

Some recent works improve the electromechanical properties of flexible composites by adding secondary fillers. Fillers with high aspect ratios or deformability such as the carbon nanotube (CNT) [26], silver nanowire (AgNW) [7], and liquid metal (LM) [15] are widely used as secondary fillers to bridge the gaps between primary fillers. They can significantly reduce the percolation threshold required for conduction and enhance conductivity. However, it remains a challenge to efficiently separate agglomerated CNTs while preventing surface damage and scission [27]. The preparation procedure of AgNW-filled composites is often complicated and accompanied by the volatilization of solvents, which is not conducive to the preparation of block samples [28,29]. The gallium oxide film on the surface of the LM droplets and the cavities formed by the droplets also weaken the electrical conductivity and mechanical strength of the composite [8]. As a component of commercial conductive silver pastes, Ag flakes are ideal secondary fillers owing to their cost-effectiveness and high conductivity. They can be overlapped, slipped, and rotated during mechanical deformation, thus connecting the primary fillers to maintain stable and robust electrical interconnects [30,31]. 

Although conductive elastomers have received extensive attention, their application as integratable sensing units in flexible electronics remains understudied. Reported flexible pressure sensors are generally of large dimensions with various shapes of block [15], film [16], and foam [32], which are difficult to be integrated into flexible electronics. Previous research demonstrated a flexible pressure-sensor array integrating 25 sensor units based on Au-coated Polydimethylsiloxane (PDMS) micropillars which showed excellent sensitivity, but the size of each sensor unit is still large (5 × 5 × 1 mm) [33]. Developing an integrated miniature sensing unit through a cost-effective and simple method remains challenging.

In this work, we report several piezoresistive composites consisting of iron powder, nickel powder, Ag flakes, and PDMS silicon rubber. To improve their sensing performance, we create conductive elastomers with anisotropic electromechanical properties through the magnetic field-assisted curing technique. They exhibit four orders of magnitude improved conductivity and significantly enhanced sensitivity along the magnetic field direction. For anisotropic elastomers filled with spike-shaped nickel powder, their resistance can be reduced by 1000 times within a tiny 2% compression, showing extremely high sensitivity. Through numerical simulations, we then analyze the conductive mechanism of the composites to explain these phenomena. Harnessing the high sensitivity of created anisotropic elastomers, we demonstrate a prototype of an integratable miniature sensor unit with a size of only 2 × 2 × 1 mm and an electronic skin (E-skin) with a 5 × 5 sensing array to accurately capture pressure signals.

## 2. Materials and Methods

### 2.1. Materials and Fabrication of Elastic Composites

The Ecoflex™ 00-30 Addition Cure Silicone Rubber was purchased from Smooth-On, Inc., Macungie, PA, USA. The SYLGARD^®^ 184 Silicone Elastomer Curing Agent and SYLGARD^®^ 184 Silicone Elastomer Base were purchased from Dow Corning, Carrollton, KY, USA. The carbonyl iron powder (2–5 µm in diameter) and Ag flake (thickness of hundreds of nanometers, average diameter of 10 µm) were purchased from Sigma-Aldrich, Melbourne, Australia. The Ni powder (3–4 µm in diameter) was purchased from Vale, Toronto, Canada. Microcontroller Arduino UNO was purchased from Jaycar, Wollongong, Australia.

Fabrication of P-Ag_x_: The elastomers filled only with Ag flakes are denoted as P-Ag_x_, where x represents the mass ratio of Ag to PDMS. In the typical method, 1 g PDMS (PDMS base/curing agent mass ratio of 9:1) was mixed in a transparent plastic cup. x represents the mass ratio of Ag flakes/PDMS in the sample (i.e., P-Ag_2.6_ means that the mass of silver flakes in this sample is 2.6 g). The weighted Ag flake was added to the mixture and stirred with a wood rod at a speed of 200 rpm for 5 min. The mixture was then poured into a mold (5 × 5 × 5 mm) and degassed under vacuum for 10 min. The vacuumed mixture was cured in an oven at 80 °C for 6 h to obtain the sample. As shown in the SEM image of P-Ag_x_ in Figure S1, the silver flakes with a high concentration are closely arranged. Due to the high aspect ratio of silver flakes, both rotation and twisting affect their spatial three-dimensional structure, which also leads to fluctuations in the electrical conductivity of the composites filled with silver flakes during deformation (see detailed discussion in Section 3.1 and Figure S1).

**Figure 1:**
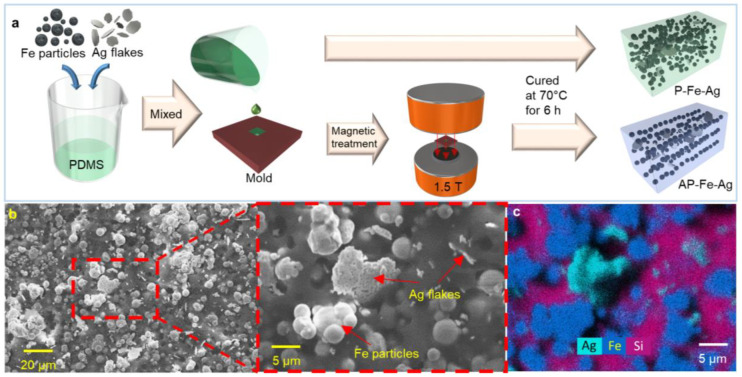
(**a**) Schematic of the preparation method of P-Fe-Ag_y_ and AP-Fe-Ag_y_. (**b**) Scanning electron microscopy (SEM) images of P-Fe-Ag_5_ and the zoom-in image to show the shape of Ag flake, (**c**) and its energy dispersive X-ray spectroscopy (EDS) analysis of the cross-section.

Fabrication of P-Fe-Ag_y_: The hybrid composites filled with iron powder and Ag flakes are denoted as P-Fe-Ag_y_, where y represents the percent weight of Ag flakes in the elastomer. The fabrication of the P-Fe-Ag_y_ is illustrated in Figure 1a. In total, 1.8 g PDMS base, 0.2 g curing agent, and 8 g iron powder (mass ratio: 0.9:0.1:4) were mixed in a transparent plastic cup. y is the mass percentage of Ag in the elastomer, which is set to 0, 1%, 1.5%, and 2.5% in this work. The mixture was then stirred, molded, and cured in the same method as P-Ag_x_. Figure 1b,c shows the SEM images and EDS analysis of the sample cross-section, which visually show the dimensions of the conductive fillers and their distribution in the elastic matrix. Smooth spherical iron particles are uniformly distributed in the PDMS elastomer to form a conductive network, while silver flakes with a high aspect ratio are randomly distributed in the PDMS matrix with different tilt angles, acting as conductive bridges to further reduce the resistivity.

Fabrication of AP-Fe/Ni-Ag_y_: The magnet-induced composites with aligned iron/nickel particles and Ag flakes are denoted as AP-Fe/Ni-Ag_y_, where y represents the percent weight of Ag flakes in the elastomer. The fabrication of the AP-Fe-Ag_y_ is illustrated in Figure 1a. The high content of iron/nickel particles will increase the viscosity of the uncured composite mixture and hinder the filler alignment in the magnetic field. Therefore, we adjusted the mass ratio of the PDMS and iron/nickel powder to 1:2.5. The preparation procedure of AP-Fe/Ni-Ag is almost the same as that of P-Fe-Ag. The only difference is that the AP-Fe/Ni-Ag mixture poured into the mold (9.6 × 9.6 × 6.2 mm) was placed in a uniform magnetic field (1.5 T) for 2 h before curing in the oven to ensure the good alignment of the Fe particles in the direction of the magnetic field.

The fabrication and application of an E-skin with AP-Ni-Ag based sensor array: The transparent and flexible E-skin was fabricated by the following steps. First, the PDMS (4 g) was spin-coated onto a silicon wafer at a speed of 500 rpm for 20 s to obtain a thin PDMS encapsulation layer (thickness of 200 μm). Second, 7 g Ecoflex (mixed with 3.5 g A liquid and 3.5 g B liquid) was poured into a mold to fabricate a middle layer with dimensions of 25.5 × 25.5 × 1 mm which has 5 × 5 pixel holes. Each pixel is filled with an AP-Ni-Ag_1_ sensing unit (2 × 2 × 1 mm). Next, 5 ultra-fine silver foil wires (500 × 1 × 0.1 mm) were pasted horizontally to the middle of each sensing unit with conductive glue to connect the sensing units in each row. The same number of silver foil wires were pasted to the reverse side of the middle layer vertically to connect the sensing units of each column. The two PDMS encapsulation layers were treated with oxygen plasma for 2 min and immediately bonded to the upper and lower surfaces of the middle layer. The device was then placed in an 80 °C oven for 60 s.

### 2.2. Experiment Equipment and Instruments

A VLS2.30 Laser Cutter (Universal Laser Systems, Inc., Scottsdale, AZ, USA) and an Original Prusa i3 MK3S+ 3D printer (Prusa Research a.s., Partyzánská, Czech Republic) were used to prepare the molds. A JEOL JSM-6490LA scanning electron microscope (JEOL, Eching b. München, Germany) was used to obtain the SEM and EDS images. An MTS Landmark 370.02 hydraulic load frame (MTS Systems, Chatswood, Australia) was used to measure the stress–strain curves of the composites. A Screw Driven Linear Guide (New Era Pump Systems, Inc., Farmingdale, NY, USA) was used to compress the samples. A MODEL WS-650MZ-23NPP spin coater (Laurell Technologies Corporation, North Wales, PA, USA) was used to prepare the PDMS film. A HARRICK PLASMA CLEANER (Harrickplasma.com., Ithaca, NY, USA) was used for oxygen plasma treatment on the PDMS film. A BUCHI V-500 (Buchi AG, Flawil, Switzerland) was used to vacuum the samples. An S&T magnetic field generator (S&T TECH. INC., Beijing, China) was used to apply a uniform magnetic field on composites. A VICI Digital Multimeter VC8145 (Shenzhen Vicimeter Technology Co., Ltd., Shenzhen, China) with a resistance range of 80 MΩ was used to measure the resistance of the samples. 

## 3. Results and Discussion

### 3.1. Electrical Property of P-Fe-Ag_y_

**Figure 2:**
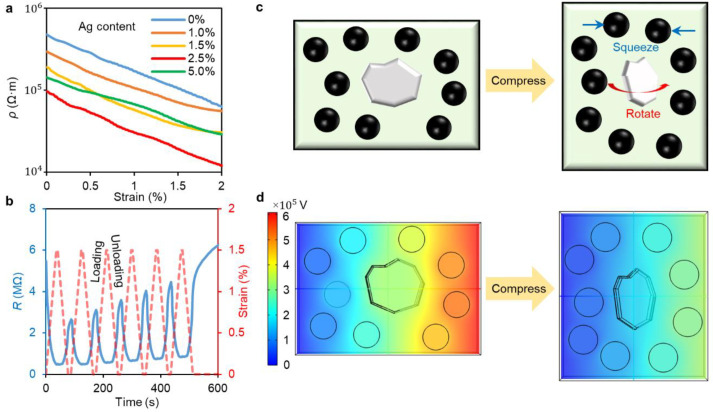
(**a**) Resistivity-strain curves of P-Fe-Ag_y_ under compression. (**b**) Resistance-strain curve of P-Fe-Ag_2.5_ under cyclic compression. The sample is pre-compressed at 0.5% strain and cyclic compressed further 1.5% strain. (**c**) Schematic diagram of microstructure changes during sample compression. (**d**) Numerical simulation of the changes in mechanical and electrical properties of samples before (**left**) and after (**right**) compression.

Figure 2a shows the resistivity-strain curves of iron particle-filled PDMS elastomers (PDMS/Fe mass ratio of 1:4) with different Ag contents. The resistivity of all samples decreases during compression. Composites with Ag mass fraction above 10% remain insulating (>80 MΩ) at 5% compressive strain. We speculate that this is because the high concentration of conductive fillers disrupts the crosslinking of the PDMS matrix, leading to incomplete curing and poor electrical conductivity. This explains why the resistivity of P-Fe-Ag_5_ is higher than that of P-Fe-Ag_2.5_, and nearly overlaps with that of P-Fe-Ag_1.5_. P-Fe-Ag_2.5_ shows the lowest initial resistivity and an order of magnitude decrease in resistivity at 2% compressive strain. Figure 2b shows the strain response of P-Fe-Ag_2.5_ under cyclic compression with 1.67% min^−1^. Due to the viscoelasticity of PDMS, there is little hysteresis in the strain response of the composite, which results in a lag of 1.75 s in the resistance change relative to the strain change. Additionally, the filler network gradually recovers to the original structure after compression, represented by the slow recovery of the maximum resistance under cyclic compression.

Figure 2c explains the piezoresistive effect of P-Fe-Ag_y_. The Fe particles and Ag flakes are separated by the PDMS layers. During the compression of the composite, they do not deform but squeeze the PDMS layers due to their rigidity. The thin Ag flake with a high aspect ratio rotates in compression and contact surrounding Fe particles, creating conductive paths and thus enhancing the conductivity. To simulate the change of resistivity during compression, we establish a 3D model of P-Fe-Ag_y_ (35 × 25 × 10 µm) and import it into COMSOL for finite element simulation. Poisson’s ratio of the PDMS matrix is set at 0.5 [15]. In the simulation, the left side of the model is set to ground and a current (0.01 A) is set to pass through the model from right to left; the other sides are set to be electrically isolated. According to the simulation results, the resistance of the model decreases from 60.9 MΩ to 34.2 MΩ when compressed by 25%. Since the resistivity of PDMS is much higher than that of conductive fillers, the resistance of the composite mainly depends on the thickness of the PDMS layers along the conductive pathways. Therefore, the squeezing of the metal particles decreases the PDMS layer thickness and sharply reduces the resistance of the composite. In real composites, the iron particles and silver flakes are in close proximity (Figure 1c), so the resistance drop caused by the squeezing between them is extremely significant.

### 3.2. Anisotropic Mechanical and Electrical Property of AP-Fe/Ni-Ag_y_

**Figure 3:**
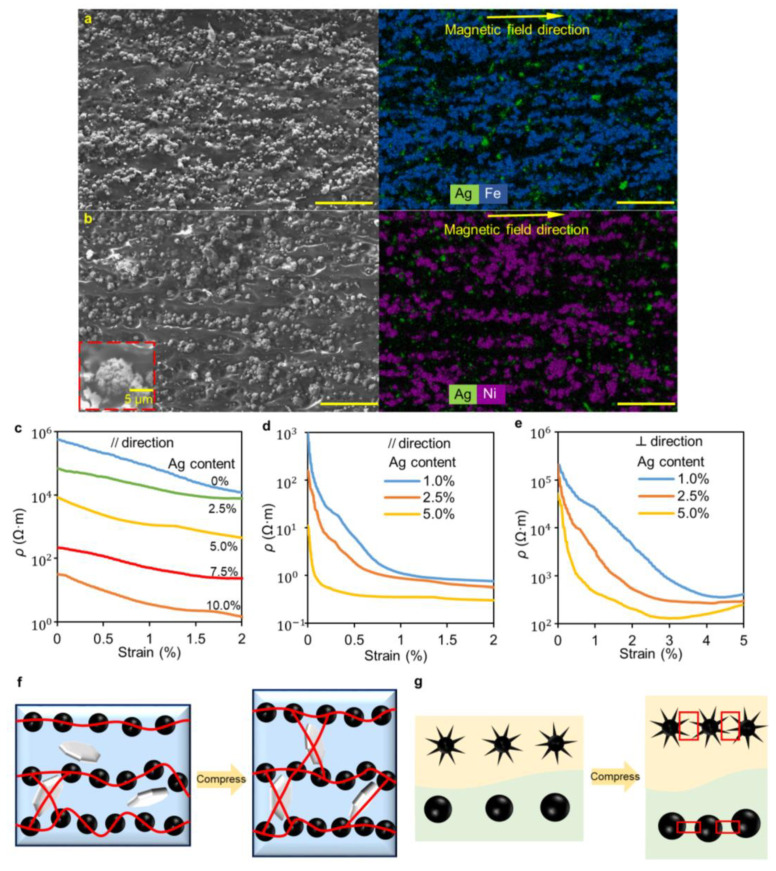
SEM images and EDS analysis of the cross-section of (**a**) AP-Fe-Ag_2.5_, and (**b**) AP-Ni-Ag_2.5_. The inset shows an SEM image of a Ni particle. Resistivity-strain curves of (**c**) AP-Fe-Ag_y_, and (**d**) AP-Ni-Ag_y_ samples under compression along the alignment direction (magnet field direction) and (**e**) AP-Ni-Ag_y_ samples perpendicular to the alignment direction. Illustrative schematics show the change in the microstructure of the (**f**) AP-Fe-Ag_y_, and (**g**) AP-Ni-Ag_y_ under compression. The scale bars are 50 μm.

Although the addition of a small number of silver flakes to the PDMS-Fe composite can significantly decrease the initial resistance, its resistivity change with strain is not obvious, indicating that its sensitivity is insufficient for application as a mechanical sensor. To improve its strain sensitivity, we create the anisotropic microstructure within the composites through the arrangement of ferromagnetic particles. After comprehensively considering the viscosity, magnetic permeability, and electromechanical properties of the composite, we set the mass ratio of PDMS and ferromagnetic powder to 1:2.5 (See detail in Figure S2). As shown in Figure 1a, the composites are cured in a uniform magnetic field to align the iron or nickel particles along the direction of the magnetic field. Figure 3a,b demonstrates the horizontal chain structures aligned by the Fe and Ni particles, which impart significant anisotropy to the composites. In addition, from the EDS analysis, the Ag flakes are sporadically distributed between the chain structures, which bridge the conductive particle chains to enhance conductivity. 

Figure 3c shows the resistivity-compressive strain curves of the AP-Fe-Ag samples along the alignment direction. The conductivity of the composites is significantly enhanced with a slight increase in the Ag mass fraction, indicating that the Ag flakes with high aspect ratios have more opportunities to contact the surrounding conductive particles to form a large number of conductive paths. Compared with P-Fe-Ag_2.5_ (Ag mass fraction of 2.5%) which has the lowest resistivity (96 kΩ·m at 0% strain) among the P-Fe-Ag composites, AP-Fe-Ag_2.5_ with the same Ag flake content and lower iron content instead shows lower resistivity (69 kΩ·m at 0% strain). This illustrates that the alignment of iron particles inside the composites along the magnetic field direction significantly increases the electrical conductivity in this direction. It should be noted that in the direction perpendicular to the magnetic field, the AP-Fe-Ag is insulated within 10% compressive strain. This is because the iron particles with high magnetic permeability are aligned into chain structures with high anisotropy. As a result, there is almost no iron particle connection between the chains; therefore, the Ag flakes with low content are unable to bridge the Fe particle chains to form sufficient conductive paths along this direction under low compressive strain.

In comparison to AP-Fe-Ag, AP-Ni-Ag exhibits higher initial conductivity and higher sensitivity due to the unique geometry of nickel particles. As shown in Figure 3d, the resistivity of the AP-Ni-Ag samples with 1% and 2.5% Ag mass fractions drops sharply by three orders of magnitude at a compressive strain of only 1%. In contrast, although the AP-Ni-Ag_5_ has the highest initial conductivity and sensitivity, its resistivity tends to stabilize prematurely when the compressive strain exceeds 0.3%. This is because the number of conductive pathways inside the samples containing high concentrations of silver flakes rapidly increases and saturates upon compression. For the application of the composite in a compression sensor, we expect it to have a suitable initial resistance (~10 KΩ) and high sensitivity over a relatively wide range. When compressed by 2% along the alignment direction, the resistivity of AP-Ni-Ag_1_ decreases by three orders of magnitude from 970 Ω·m to 0.5 Ω·m, showing an excellent sensing performance in tiny strain detection. 

Figure 3e shows the resistivity-strain curves of the AP-Ni-Ag samples perpendicular to the alignment direction. The resistivity of the samples along this direction also decreases with the increase in the concentration of silver flakes. However, the resistivity in this direction is 1000 times higher than that of the same sample in the alignment direction, showing the high anisotropy of AP-Ni-Ag. Such high anisotropy avoids interference in the sensor caused by the current flow perpendicular to the alignment direction. Additionally, with the increase in compressive strain, the resistivity of the samples recovers slightly after stabilization, reflecting the destruction after saturation of conductive networks (see details in Figure S3). The concentration of nickel particles in the conductive paths perpendicular to the alignment direction is low. After the conductive path is saturated, further compression of the sample will result in its horizontal expansion and horizontal separation of the nickel particles, thereby disrupting the conductive paths and increasing the resistivity. In addition to the anisotropic electrical properties, the AP-Fe/Ni-Ag_y_ samples also exhibit markedly anisotropic mechanical properties (see the results of the mechanical tests in Figure S4). Their elastic modulus along the alignment direction is about 2.2 times that along the perpendicular direction.

Figure 3f,g shows the illustrative schematics of the conductive filler networks in the composites and their spatial change of location under compression. In Figure 3f, without external stress, the conductive paths inside the sample consist of aligned particle chains and Ag flakes bridging them. When the sample is compressed in the alignment direction, the PDMS layers between the metal particles are compressed, thus reducing the resistance of the particle chains. The slip and rotation of the Ag flakes during compression create more conductive paths to further improve the conductivity of the sample. In recent years, quantum tunneling between irregular conductive particles in elastomeric matrices has been proposed in several studies [34,35]. Unlike percolation threshold theory, which focuses on the static conductivity of composites, quantum tunneling effects are commonly used to explain the exponential change in the resistance of composites under deformation. The elastomer filled with irregular conductive particles shows a faster resistance decline under compression than that filled with smooth spherical particles, as shown in Figure 3g. The inset in Figure 3b shows an SEM image of a Ni particle. The synaptic structures on the surface of irregular nickel particles make it easier to contact adjacent conductive fillers under strain, significantly increasing the conductivity of the composites. In contrast, smooth iron particles require greater compression to contact to form conductive pathways. Therefore, composites filled with spike-shaped nickel particles exhibit higher sensibility in comparison to that filled with smooth iron particles.

### 3.3. Applications of AP-Ni-Ag_y_

**Figure 4:**
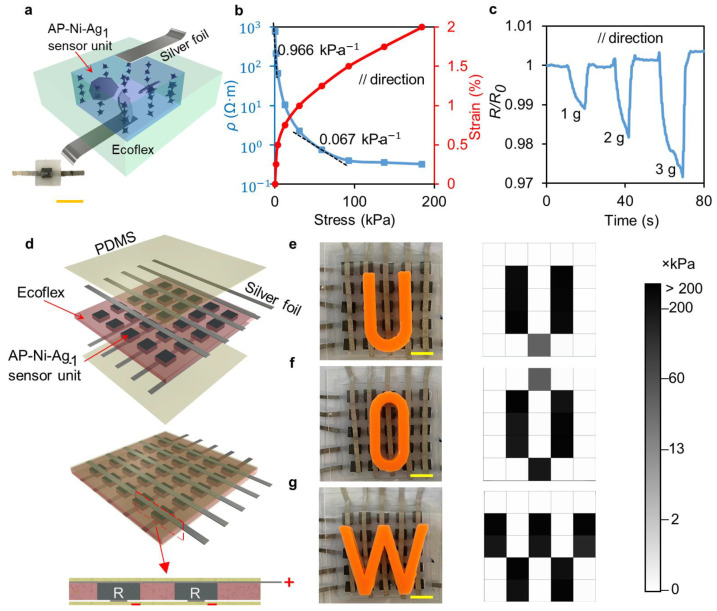
(**a**) Schematic diagram and real image of the AP-Ni-Ag_1_ sensor unit. (**b**) The resistivity–stress curve of the sensor unit under 2% compression strain along the direction of magnetic field lines. (**c**) Relative resistance change of the sensor unit with the load of 1 g, 2 g, and 3 g. (**d**) Schematic diagram of E-skin with AP-Ni-Ag_1_-based 5 × 5 sensor array. Real images of the E-skin with human loading on (**e**) “U”, (**f**) “O”, and (**g**) “W” letter blocks and their corresponding grey-scale pressure pattern. The scale bars are 5 mm.

Harnessing the extremely high sensitivity of the anisotropy composite, we demonstrate its application as a simple pressure sensor unit that can be integrated into flexible devices. The main component of the sensor is an AP-Ni-Ag_1_ sample (2 × 2 × 1 mm) with silver foil electrodes on the top and bottom, which is embedded in an Ecoflex interlayer and encapsulated by two thin PDMS layers (see Figure 4a). Young’s modulus of the AP-Ni-Ag_1_ is much higher than that of the Ecoflex interlayer, which allows the sensor to concentrate pressure on the composite when it is compressed, thus improving the pressure sensitivity. The AP-Ni-Ag_1_ sample is connected to the circuit along the alignment direction. Figure 4b shows the resistivity–stress curve of the AP-Ni-Ag_1_ sensor under compression. The pressure sensitivity (S) is defined by Equation (1):

(1) $$S=\frac{\frac{\Delta \rho }{\rho }}{\Delta P}$$

where, Δ*ρ* is the change in the resistivity, *ρ* is the resistivity of the sensor, and Δ*P* is the change in the applied pressure. The sensitivity of the sensor unit is highly affected by the pressure. Under the pressure of 1.6 kPa, the sensor is compressed by 0.25% with a resistivity decline of 3.7 times, exhibiting high-pressure sensitivity of 0.966 kPa^−1^. As the pressure increases more than 60 kPa, the sensor sensitivity gradually decreases to 0.067 kPa^−1^. To determine the detection limit of the sensor unit, we load PDMS cubes weighing 1, 2, and 3 g onto it. The relative resistance changes are approximately 1%, 2%, and 3%, respectively, demonstrating the low detection limit and high-pressure resolution of the sensor (Figure 4c). In addition, the sensor unit also shows high stability in the cyclic test under different pressures. The sensor is repeatedly loaded with weights weighing 50 g, 100 g, and 200 g, and all of them show a large resistance change. The resistance of the sensor rises with the number of cyclic loadings, which indicates that some of the conductive paths in the sample are disrupted under cyclic loading, but the resistance change tends to be stable after multiple compressions (see details in Figure S5). Owing to the PDMS encapsulation layer, the sensor unit can be prevented from the humid environment. As the ambient temperature rises to 50 °C, the initial sensitivity of the sensor unit is only reduced by 0.8% to 0.958 kPa^−1^, indicating a stable sensing performance within the normal ambient temperature range (see details in Figure S6). With high sensitivity, a wide sensing range, flexible environmental adaptability, and small size, the AP-Ni-Ag_1_ sensor unit shows advantages in applications in integratable flexible electronics.

Based on the integratable miniature sensor units with excellent sensing performance in a tiny strain range, we develop an E-skin with the AP-Ni-Ag_1_-based sensor array. Figure 4d shows the 3D structure diagram and exploded schematics of the E-skin. Twenty-five sensor units are integrated into a 25.5 × 25.5 mm Ecoflex substrate, enabling high spatial resolution in a small area. The E-skin is connected to a microcontroller for the real-time display of pressure distribution, as shown in Figure 4e–g. When the E-skin is pressed by the subject’s finger or loaded with objects, the real-time pressure distribution map calculated from the variation of the current through the sensor units caused by their resistance change is presented in Figure 4e–g and Video S1. When pressing the letter blocks placed on top of the E-skin, the high-resolution E-skin can recognize the outline of the objects. The distribution and magnitude of the pressure can be clearly observed from the map, and the sensing units around the load area are not erroneously affected, showing high accuracy. Interestingly, some tiny areas of the sensor units in Figure 4e–g are covered by letter blocks, but they are not compressed when applying human loading on the letter blocks. These sensor units are forced out of the stress area because the Ecoflex extends laterally under pressure, resulting in no electrical signal on the pressure pattern. The time required to sample and process the electrical signals of 25 sensors is only 144 ms, demonstrating the fast response of the E-skin. Such a small-scale sensor array with real-time response and high sensitivity is of great significance in signal detection, human–machine interface, and prosthetic devices.

## 4. Conclusions

In this study, we developed several PDMS-based composites filled with Ag flakes and ferromagnetic particles. In addition to reducing the resistivity of the composites using high-aspect-ratio Ag flakes, we also created anisotropic composites through a magnetic field-assisted alignment technique to significantly improve their electrical conductivity and sensitivity. Along the alignment direction, AP-Fe-Ag_y_ exhibits a much higher elastic modulus, conductivity, and sensitivity. In addition, the quantum tunneling effect caused by the spike-shaped Ni particles further improves the conductivity and sensitivity of the composites. The resistivity of AP-Ni-Ag_1_ drastically reduces from 970 Ω·m to 1.1 Ω·m within only 1% compressive strain. Harnessing the excellent electrical properties of AP-Ni-Ag, we demonstrate an integratable sensor unit with a dimension of only 2 × 2 × 1 mm. It shows superior sensing performance with a high-pressure sensitivity of 0.966 kPa^−1^ and a wide sensing range of up to 200 kPa. Based on this sensor unit, we develop an electronic skin with a 5 × 5 sensor unit array for high-resolution real-time pressure distribution detection. We believe that this pressure sensor with a small scale, high resolution, rapid response, and high sensitivity is competent for various applications in stretchable electronics including signal detection, human–machine interface, and prosthetic devices.

## Data Availability

The authors declare that the main data supporting the findings of this study are available within the article and its Supplementary Materials. Extra data are available from the corresponding author upon reasonable request.

## Supplementary Materials

The following supporting information can be downloaded at: https://www.mdpi.com/article/10.3390/nano12224018/s1, Figure S1: The SEM, EDS, and electrical properties of P-Ag_x_; Figure S2: The mechanical and electrical properties of AP-Ni_z_; Figure S3: Schematic diagram of the AP-Ni-Ag_y_ when compressed perpendicular to the direction of magnetic field lines; Figure S4: Strain–stress curves in two directions of AP-Fe/Ni-Ag_y_; Figure S5: The strain response characterization of the AP-Ni-Ag_1_ sensor for the cyclic compression test with three load levels; Figure S6: The resistivity-stress curve of sensor unit under 2% compression strain along the direction of magnetic field lines at 50 °C; Video S1: Real-time monitoring of several pressure signals.
