# Supplementary material for: Silver Nanoflakes-Enhanced Anisotropic Hybrid Composites for Integratable Pressure Sensors
Source: Nanomaterials (Basel). 2022 Nov 16;12(22):4018. doi: 10.3390/nano12224018 (PMC9698126; doi:10.3390/nano12224018)
Supplement: Supplementary file 1 [file nanomaterials-12-04018-s001.zip › Supplymentary.pdf]

Supporting Information

Silver Nanoflakes-Enhanced Anisotropic Hybrid Composites for Integratable Pressure Sensors

Qingtian Zhang [1], Guolin Yun [2,*], Shida Jin [1], Zexin Chen [1], Shi-Yang Tang [3], Hongda Lu [1], Haiping Du [4] and Weihua Li [1,*]

[1] School of Mechanical, Materials, Mechatronic and Biomedical Engineering, University of Wollongong, Wollongong, 2522, Australia
[2] Cambridge Graphene Centre, University of Cambridge, Cambridge CB3 0FA, UK
[3] Department of Electronic, Electrical and Systems Engineering, University of Birmingham, Edgbaston, Birmingham B15 2TT, UK
[4] School of Electronic, Computer and Telecommunications Engineering, University of Wollongong, Wollongong 2522, Australia
* Correspondence: gy264@cam.ac.uk (G.Y.); weihuali@uow.edu.au (W.L.)

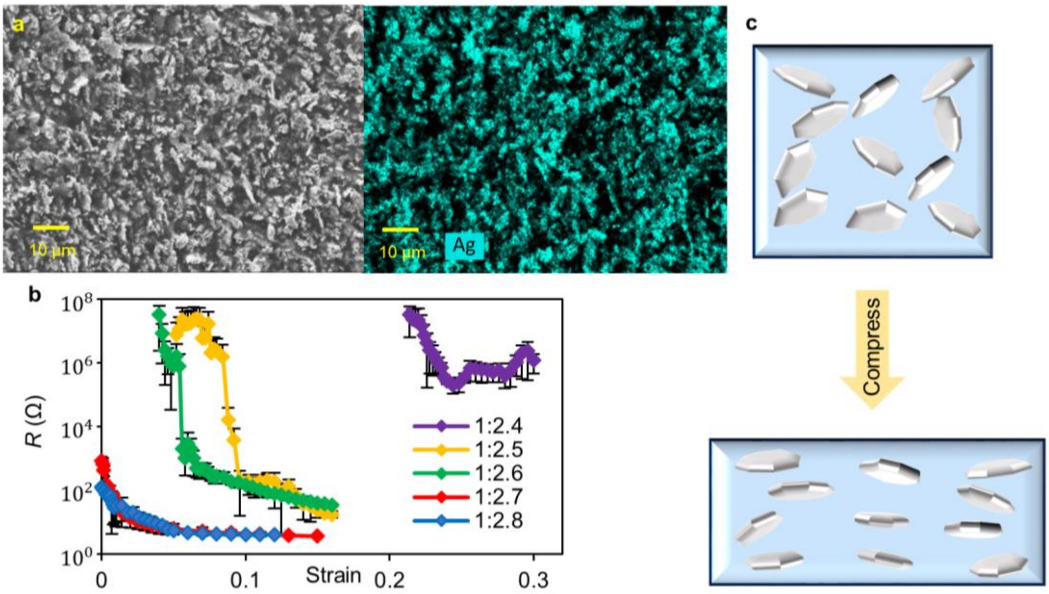

Figure S1. (a) SEM images of P-Ag_{10} and its EDS analysis of the cross section. (b) Resistance-strain curves of P-Ag_x under compress test. The values of the error bars are the standard deviation of the modulus under 3 measurements. (c) Schematic diagram of the movement of Ag flakes inside the P-Ag_x during compression.

We mixed Ag flakes as a single filler with PDMS elastomer according to the different mass ratios to obtain several P-Ag_x composites. In Figure S1b, as the Ag flake content rise, the initial resistance of the composites decrease. When the PDMS to Ag flake mass ratio reaches 1:2.7, the initial resistance of the composite decreases to 1000 Ω. We judged that the Ag flake concentration reached the percolation at that time. We observed that the resistance sharply dropped at the beginning of compression, and trended to be stable in further compression. This is because the squeeze of the PDMS layer by the Ag flakes in compression reduces the thickness of the insulation layer, significantly improving the conductivity. In addition, the flip and slip of the Ag flakes have an impact on the generation of conductive paths. When Ag flakes are squeezed to touch each other, no more conductive paths can be created and the resistance remains stable. However, when the filler concentration of the composite is low, further compression results in a rise in resistance. For P-$Ag_{2.4}$, the resistance displayed a slight rise after 0.25 compression strain. We give a hypothesis that a low filler content in composite leads to an uncompacted distribution of Ag flakes. Therefore, at compressing the composite the Ag flakes in the PDMS matrix are dragged along with the polymer chains and pulled apart, which decreases the number of conductive paths and the temporary conducting network tends to collapse, which is shown in Figure S1c.

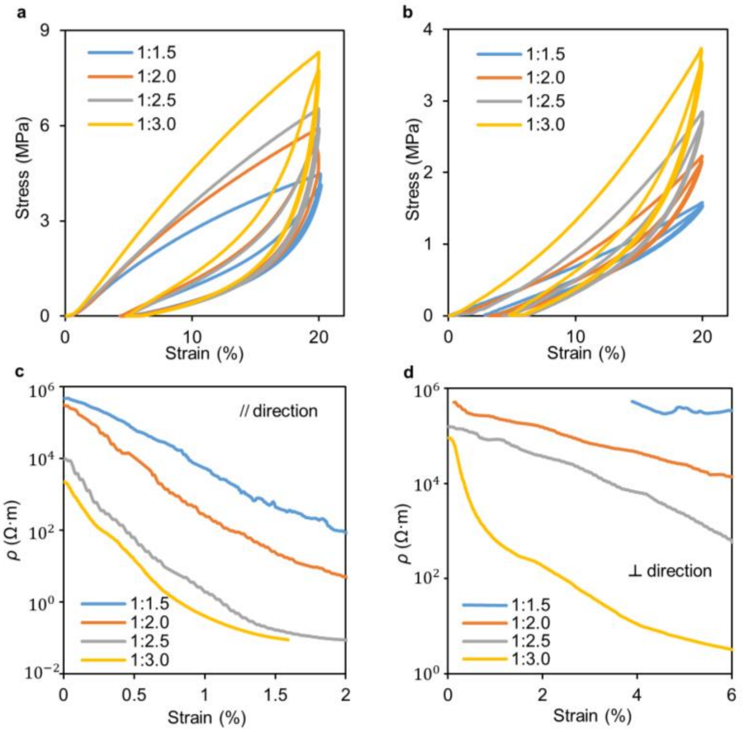

Figure S2. Strain-stress curves of AP-Ni_z (a) along the magnet field lines and (b) perpendicular to magnet field lines in the first two compressing cycles with the speed of 1.2 mm·min-1. Resistivity-strain curves of AP-Ni_z (c) along the magnet field lines and (d) perpendicular to magnet field lines under compression test.

We prepared samples with only different nickel powder, and called it AP-Ni_z, in which z represents the mass ratio of PDMS and nickel powder. We used the experimental images above to determine the mass ratio of PDMS to Ni in the AP-Ni-Ag_y sample to be z = 1:2.5. AP-$Ni_{2.5}$ showed excellent electrical properties for the resistance can sharply drop from 105 Ω·m to 10-1 Ω·m within 2% compression strain, which is much better than AP-$Ni_{1.5}$ and AP-$Ni_{2.0}$. What's more, the strain response of AP-$Ni_{3.0}$ was a little better than that of AP-$Ni_{2.5}$ but the stress reached 8.31 MPa at 20% compression strain which is much higher than that of AP-$Ni_{2.5}$ (6.52 MPa). Most interestingly, when z = 3.0, the anisotropic property of the sample after magnetic field induction becomes smaller because the concentration of nickel powder in the sample is too high. This is reflected in the fact that the initial resistance of the sample and the trend of decreasing resistance are approximately the same in the measurement directions parallel and perpendicular to the magnetic field lines. After weighing the material conductivity, sensitivity and Young's modulus, we finally chose z = 2.5 for the preparation of anisotropic elastomers containing silver and nickel powder.

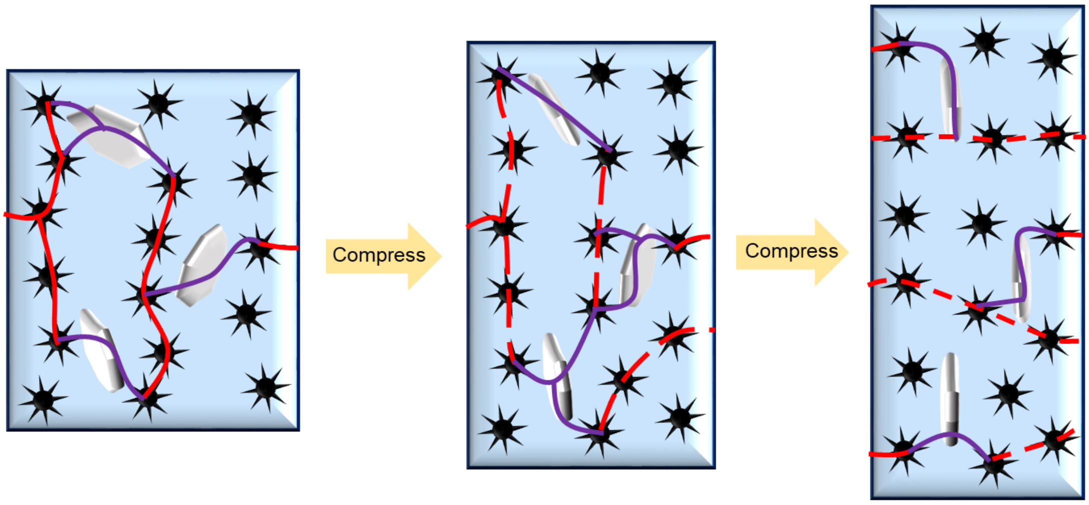

Figure S3. Schematic diagram of the AP-Ni-Ag_y when compressed perpendicular to the direction of magnetic field lines. The red solid line represents the conductive paths between Ni particles when they are in close proximity. The red dashed line indicates the conductive paths between the Ni particles when they are separated. The solid purple line indicates the conductive paths between the Ni particles and the Ag flakes.

At the original state, the Ag flake acts as a conductive bridge within the composite connecting adjacent chains of Ni particles. After primary compressing, the rotation of the Ag flakes and the proximity of the Ni particle chains due to Poisson's ratio of 0.5 allows the generation of more conductive paths inside the composite, indicating the reduction of resistance. At the same time, the Ni particles on the chains display a slight separation, which weakens the conductive ability of the paths. With further compression, the Ni particles that are in the chain are not tunneled due to the large distance, reducing the conductive paths.

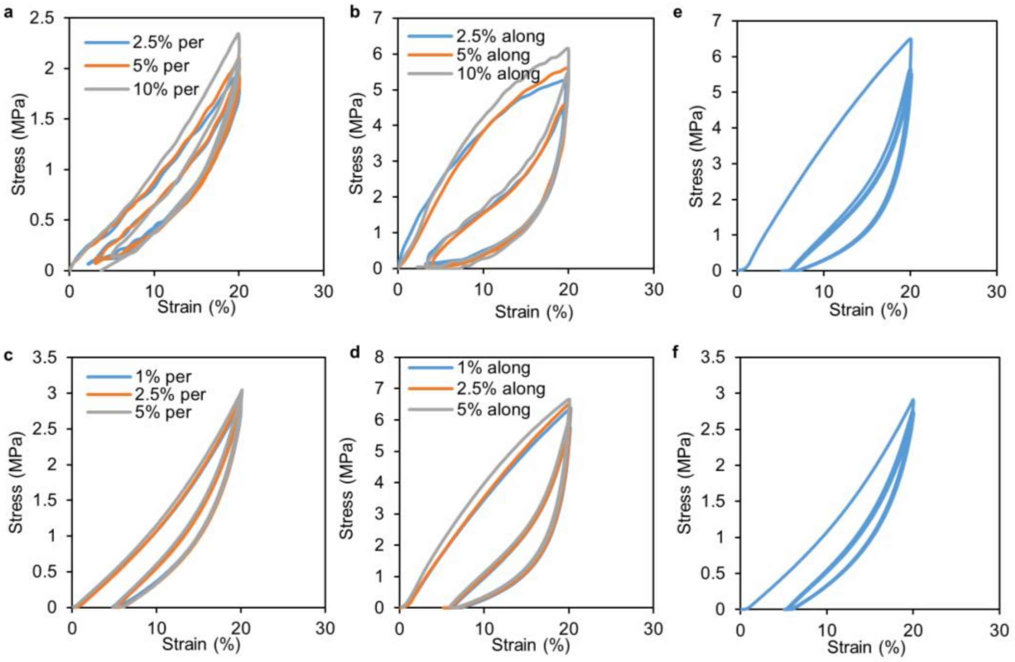

Figure S4. Strain-stress curves in two directions (perpendicular to and along the direction of the magnet field lines) of (a, b) AP-Fe-Ag$_y$, and (c, d) AP-Ni-Ag$_y$ under 20% compression with the speed of 1.2 mm·min-1. (e, f) Strain-stress curves in two directions of AP-Ni-Ag$_{2.5}$ under cyclic loading test.

To investigate the anisotropic properties of magnetorheological elastomers induced by magnetic fields, we performed stress-strain experiments and strain-response experiments along perpendicular and parallel magnetic field lines. The AP-Ni-Ag$_y$ were compressed 20% strain along parallel and perpendicular directions, which y = 1, 2.5, and 5. The curves demonstrated elastic-plastic behavior similar to rubber, which had an area surrounded by loading and unloading lines. For AP-Fe-Ag$_5$, the maximum stress at 0.2 strain is about 6.65 MPa at the parallel magnetic field lines direction, while the value is 3.04 MPa at the perpendicular magnetic field lines direction, indicating an obvious mechanical anisotropy phenomenon. We also conducted the compression test in more than 3 cycles, and the stress value of 20% compression strain decreased with an increasing number of cycles due to the hysteresis of the viscoelastic composites under external pressure. What's more, the area of the hysteresis region is greatly reduced compared to the first circle, and basically remained stable in the subsequent cycles, indicating the property of stress softening. The AP-Fe-Ag$_y$ samples showed similar properties as the AP-Ni-Ag$_y$. The maximum stress of AP-Fe-Ag$_{2.5}$ at 0.2 strain is about 5.21 MPa in the parallel magnetic field lines direction, while the value is 1.92 MPa in the perpendicular magnetic field lines direction. In conclusion, the AP-Fe/Ni-Ag$_y$ have the classic mechanical properties of soft rubber elastomers, and show large differences in maximum stress and viscoelasticity along and perpendicular to the direction of the magnetic lines.

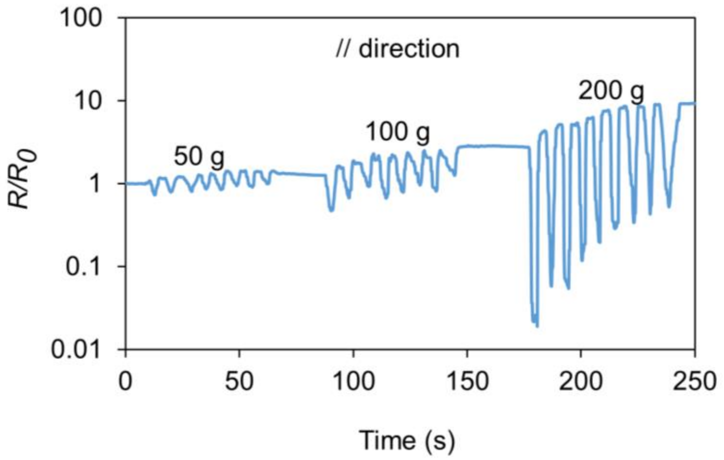

Figure S5. The strain response characterization of AP-Ni-Ag_1 sensor for the cyclic compression test with three load levels.

The resistance change curve of the sensor unit in the cyclic test under different pressures (weights of 50, 100, and 200 g) is shown in Figure S5. Each weight is placed on the sensor for 3 seconds and then removed for 3 seconds, repeating 8 cycles. Under pressure, the resistance reduction of the sensor doubled from 33% under a load of 50 g to 63% under a 100 g load. When the load increases to 200g, the resistance drops drastically by 143 times, showing an excellent sensitivity of the sensor unit. The resistance of the sensor rises with the number of cyclic loadings, which indicates that some of the conductive paths in the sample are disrupted under cyclic loading, but the resistance change tends to be stable after multiple compressions.

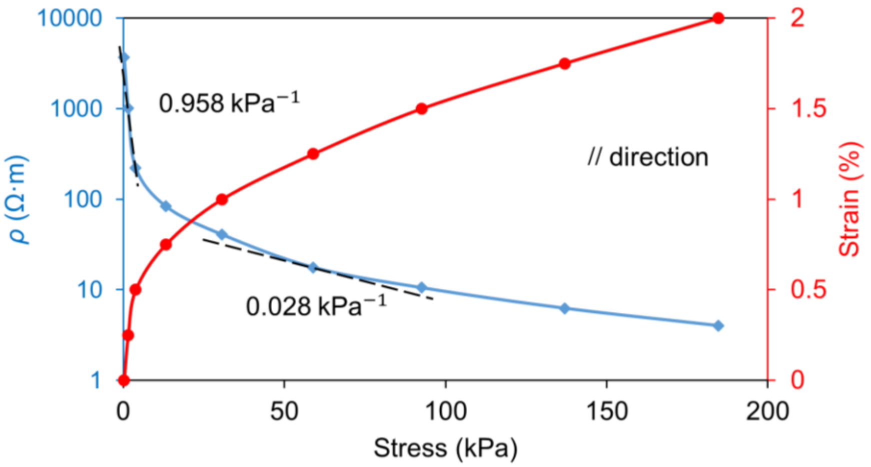

Figure S6. The resistivity-stress curve of sensor unit under 2% compression strain along the direction of magnetic field lines at 50 °C.

As shown in figure, the higher temperature will increase the resistivity of the sensor unit because of the volume expansion of the composite. The thickness of the insulating elastic layer between the fillers is increased, resulting in fewer conductive paths inside the composite. However, the sensor unit still maintains high pressure sensitivity of 0.958 kPa^{-1} under a pressure of 1.5 kPa, which is only 0.8% different from its sensitivity at room temperature (0.966 kPa^{-1}). The resistance increase of the sensor caused by the rising ambient temperature can be neglected in comparison to the exponential decline of the resistance under mechanical deformation. Therefore, the device can maintain normal working performance within the normal ambient temperature range.
